# Correction: Laniecki, R.; Magowski, W.L. New Definition of *Neoprotereunetes* Fain et Camerik, Its Distribution and Description of the New Genus in Eupodidae (Acariformes: Prostigmata: Eupodoidea). *Animals* 2023, *13*, 2213

**DOI:** 10.3390/ani13243761

**Published:** 2023-12-06

**Authors:** Ronald Laniecki, Wojciech L. Magowski

**Affiliations:** Department of Animal Taxonomy and Ecology, Adam Mickiewicz University, Uniwersytetu Poznańskiego 6, 61-614 Poznań, Poland; ronald.laniecki@amu.edu.pl

Authors’ address correction:

The authors’ address area code is misspelled: it is «61-714», while it should be «61-614».

Text Correction:

There was an error in the original publication [1]. The new genus-level taxon that was created, namely, *Antarcteupodes* Laniecki gen. nov., has not been registered in Zoobank, and consequently, the Zoobank accession number has not been conferred. 

A correction has been made:

The Zoobank accession number below is now associated with the new scientific name *Antarcteupodes*:

urn:lsid:zoobank.org:act:515FA061-406E-46E6-A605-91912AEF714E.

The authors state that the scientific conclusions are unaffected. This correction was approved by the Academic Editor. The original publication has also been updated.
